# Supplementary material for: Observation of spatiotemporal optical vortices enabled by symmetry-breaking slanted nanograting
Source: Nat Commun. 2024 Apr 9;15:3055. doi: 10.1038/s41467-024-47475-2 (PMC11003997; doi:10.1038/s41467-024-47475-2)
Supplement: Supplementary file 1 — Supplementary Information [file 41467_2024_47475_MOESM1_ESM.pdf]

Supplementary Materials for

Observation of spatiotemporal optical vortices enabled by symmetry-breaking slanted nanograting

Pengcheng Huo[1,2,#], Wei Chen[1,2,#], Zixuan Zhang[3,#], Yanzeng Zhang[1,2], Mingze Liu[1,2], Peicheng Lin[1,2], Hui Zhang[1,2], Zhaoxian Chen[1,2], Henri Lezec[4], Wenqi Zhu[4,5], Amit Agrawal[4], Chao Peng[3,*], Yanqing Lu[1,2,*], Ting Xu[1,2,*]

1. National Laboratory of Solid-State Microstructures, College of Engineering and Applied Sciences and Collaborative Innovation Center of Advanced Microstructures, Nanjing University, Nanjing 210093, China

2. Key Laboratory of Intelligent Optical Sensing and Manipulation, Ministry of Education, Nanjing University, Nanjing 210093, China

3. State Key Laboratory of Advanced Optical Communication Systems and Networks, School of Electronics, Frontiers Science Center for Nano-optoelectronics, Peking University, Beijing 100871, China

4. National Institute of Standards and Technology, Gaithersburg, Maryland 20899, USA

5. Maryland NanoCenter, University of Maryland, College Park, Maryland 20742, USA

# These authors contribute equally to this work

[*] Email: pengchao@pku.edu.cn, yqlu@nju.edu.cn, xuting@nju.edu.cn

Section 1: Complex transmission coefficient of device

We use temporal coupled-mode theory (TCMT) [1-4] to analyze the behavior of the device discussed in the main-text. Assuming a plane wave incident on the device, in order to respect the time-reversal (TR) symmetry for a general geometry and arbitrary incident angle, we include the resonance at both $+k$ and $-k$ into the formulation, resulting in a two-resonance, four-port TCMT model:

$$\frac{dA}{dt} = (j\omega_0 - \gamma)A + K^T S_+ \tag{S1}$$

$$S_- = SS_+ = CS_+ + DA \tag{S2}$$

in which:

$$S_+ = \begin{pmatrix} S_{1+} \\ S_{2+} \\ S_{3+} \\ S_{4+} \end{pmatrix},\ S_- = \begin{pmatrix} S_{1-} \\ S_{2-} \\ S_{3-} \\ S_{4-} \end{pmatrix},\ A = \begin{pmatrix} A_k \\ A_{-k} \end{pmatrix} \tag{S3}$$

$$\mathrm{K}^T = \begin{pmatrix} \kappa_1 & 0 & \kappa_3 & 0 \\ 0 & \kappa_2 & 0 & \kappa_4 \end{pmatrix},\quad \mathrm{D} = \begin{pmatrix} 0 & d_1 \\ d_2 & 0 \\ 0 & d_3 \\ d_4 & 0 \end{pmatrix},\quad C = e^{j\phi} \begin{pmatrix} 0 & r & 0 & jt \\ r & 0 & jt & 0 \\ 0 & jt & 0 & r \\ jt & 0 & r & 0 \end{pmatrix} \tag{S4}$$

Here A are the complex amplitude of the two resonances mode at $+k$ and $-k$, respectively, ω_0 is the resonance frequency, γ is the radiation decay rate. S_+ and S_- are the amplitudes of incoming and outgoing waves. The superscript T represents the transpose of the matrix. K is the coupling matrix from the incidence to the resonance mode, D is the coupling matrix from the resonance to the outgoing wave. C is a scattering matrix describing the direct scattering process without the resonance (namely, the Fabry-Perot background), where r and t are reflection/transmission coefficients satisfying $r^2+t^2=1$. The initial phase ϕ depends on the choice of the reference plane position. Energy conservation and reciprocity constrain C to be unitary and symmetric. S is a matrix describing the scattering process with the resonance. When the resonance is excited by external incident wave with frequency ω, the scattering matrix S is written as:

$$S = [C + \frac{DK^T}{j(\omega - \omega_0) + \gamma}] \tag{S5}$$

Here the constant K, D and C are related to each other via energy conservation and TR

symmetry [5-7], followed as:

$$DD^{\dagger} = 2\gamma, \qquad CD^{*} = -K, \qquad D = K\sigma_x \tag{S6}$$

where the superscript * and † represent conjugation and Hermitian conjugation of the matrix, respectively. σ_x is the X-Pauli matrix acting to flip the resonances. And hence,

$$e^{j\phi}(rd_2^* + jtd_4^*) = -d_1\,, \qquad e^{j\phi}(jtd_2^* + rd_4^*) = -d_3 \tag{S7}$$

$$\gamma_1 + \gamma_3 = \gamma_2 + \gamma_4 = \gamma \tag{S8}$$

Combine Eq. (S5) with Eq. (S6), we get:

$$S = e^{j\phi}\begin{pmatrix} 0 & r & 0 & jt \\ r & 0 & jt & 0 \\ 0 & jt & 0 & r \\ jt & 0 & r & 0 \end{pmatrix} + \frac{1}{j(\omega-\omega_0)+\gamma}\begin{pmatrix} 0 & d_1d_2 & 0 & d_1d_4 \\ d_2d_1 & 0 & d_2d_3 & 0 \\ 0 & d_3d_2 & 0 & d_3d_4 \\ d_4d_1 & 0 & d_4d_3 & 0 \end{pmatrix} \tag{S9}$$

Without loss of generality, we fix the phase ϕ to be 0 by appropriately choosing the location of the reference plane. Substituting Eq. (S6) - Eq. (S8) into Eq. (S9), we derive the transmission coefficient of the matrix S as:

$$S_{14} = jt - \frac{d_4(rd_2^* + jtd_4^*)}{j(\omega-\omega_0)+\gamma_2+\gamma_4} \tag{S10}$$

We write the decay rates to different ports as $d_i = \sqrt{2\gamma_i}e^{j\theta_i}$, and thus we obtain the equations：

$$S_{14} = \frac{-t(\omega-\omega_0) - 2r\sqrt{\gamma_2\gamma_4}cos(\theta_4-\theta_2)}{j(\omega-\omega_0)+\gamma_2+\gamma_4} + j\frac{t(\gamma_2-\gamma_4) - 2r\sqrt{\gamma_2\gamma_4}sin(\theta_4-\theta_2)}{j(\omega-\omega_0)+\gamma_2+\gamma_4} \tag{S11}$$

According to Eq. (S7), we readily solve that:

$$sin(\theta_4-\theta_2) = \frac{\gamma_1 - r^2\gamma_2 - t^2\gamma_4}{2rt\sqrt{\gamma_2\gamma_4}} \tag{S12}$$

$$cos(\theta_4-\theta_2) = \pm\sqrt{1-(\frac{\gamma_1 - r^2\gamma_2 - t^2\gamma_4}{2rt\sqrt{\gamma_2\gamma_4}})^2} \tag{S13}$$

By substituting Eq. (S12) and Eq. (S13) into Eq. (S11), we get:

$$S_{14} = \frac{-t(\omega-\omega_0) \pm \sqrt{4r^2t^2\gamma_2\gamma_4 - (\gamma_1 - r^2\gamma_2 - t^2\gamma_4)^2}/t}{j(\omega-\omega_0)+\gamma_2+\gamma_4} + j\frac{t(\gamma_2-\gamma_4) - (\gamma_1 - r^2\gamma_2 - t^2\gamma_4)/t}{j(\omega-\omega_0)+\gamma_2+\gamma_4} \tag{S14}$$

For a given resonance, its radiation decay rates (γ_2, γ_4) are intrinsically determined by the eigenstate itself. The value of θ_4 and θ_2 can be judged by the sign of Eq.(S12). So that either positive or negative signs in Eq.(S13) and Eq.(S14) can be determined accordingly.

Section 2: Topological singularity of complex transmission coefficient

To find the topological singularity of the transmission coefficient near the resonant frequency, we express $\boldsymbol{s}$ as a complex number,

$$s = \mathrm{Re}(s) + j\mathrm{Im}(s) \tag{S15}$$

Where $\mathrm{Re}(s)$ and $\mathrm{Im}(s)$ represent the real and imaginary part of complex transmission coefficient $\boldsymbol{s}$, respectively, given by,

$$\mathrm{Re}(s) = \frac{(\gamma_2+\gamma_4)\left[-t(\omega-\omega_0) \pm \sqrt{4r^2t^2\gamma_2\gamma_4-(\gamma_1-r^2\gamma_2-t^2\gamma_4)^2}/t\right]}{(\omega-\omega_0)^2+(\gamma_2+\gamma_4)^2} + \frac{(\omega-\omega_0)[t(\gamma_2-\gamma_4)-(\gamma_1-r^2\gamma_2-t^2\gamma_4)/t]}{(\omega-\omega_0)^2+(\gamma_2+\gamma_4)^2} \tag{S16}$$

$$\mathrm{Im}(s) = \frac{(\gamma_2+\gamma_4)[t(\gamma_2-\gamma_4)-(\gamma_1-r^2\gamma_2-t^2\gamma_4)/t]}{(\omega-\omega_0)^2+(\gamma_2+\gamma_4)^2} + \frac{(\omega-\omega_0)\left[-t(\omega-\omega_0) \pm \sqrt{4r^2t^2\gamma_2\gamma_4-(\gamma_1-r^2\gamma_2-t^2\gamma_4)^2}/t\right]}{(\omega-\omega_0)^2+(\gamma_2+\gamma_4)^2} \tag{S17}$$

Accordingly, the phase of complex transmission coefficient $\boldsymbol{s}$ is derived as

$$\varphi(s) = \mathrm{atan}\frac{\mathrm{Im}(s)}{\mathrm{Re}(s)} \tag{S18}$$

In the main-text, we focus on the 2D frequency-momentum space (ω-k_x). When k_x and ω continuously varies from point P^1 to P^2, the phase Ψ accumulated along the path $P^1 \rightarrow P^2$ in the complex plane $[\mathrm{Re}(s), \mathrm{Im}(s)]$ is expressed as,

$$\Psi = \int_{\varphi[s(P^1)]}^{\varphi[s(P^2)]} d\varphi \tag{S19}$$

In the ω-k_x space, the transmission coefficient s nullifies at the winding center of the phase spiral, namely $s(P^s) = \mathrm{Re}[s(P^s)] + j\mathrm{Im}[s(P^s)] = 0$, we refer to such a point P^s as zero-valued singularity. If an isolated singularity point P^s appears, the accumulated phase along any closed path $\mathbb{C}$ encircling the P^s is $\Psi = \oint d\varphi = 2\pi$, and the winding number of the path $\mathbb{C}$ around the P^s is given by,

$$l = \frac{\Psi}{2\pi} = 1 \tag{S20}$$

Therefore, the zero-valued singularity exhibits phase vortex of topological charge l=1 in the transmission of device. Conversely, if there is no isolated singularity point P^s in ω-k_x space, the texture of the complex transmission does not contain any notable feature about STOV.

According to Eq(S16) and Eq(S17), the necessary condition of zero-transmission in the ω-k_x space can be derived from $\mathrm{Re}[S_{14}] = \mathrm{Im}[S_{14}] = 0$ as

$$\begin{cases} (\omega - \omega_0)^2 = \dfrac{4r^2\gamma_2\gamma_4}{t^2} - (\gamma_2 - \gamma_4)^2 \\ \gamma_1 = \gamma_2 \\ t \neq 0 \end{cases} \tag{S21}$$

In our case, the condition S21.3 ($t \neq 0$) always holds. The condition S21.1 describes the coupling between the background and the radiation from the resonance itself. It's obviously that in the premise of the frequency deviation, the condition S21.1 can also be fulfilled: for a specific resonance at k_x, the mismatch between the background and the radiation can be compensated by the frequency detuning $\omega - \omega_0$. Moreover, considering that we have $r^2 + t^2 = 1$, the degree of freedom (DoF) of the condition S21.1 is exactly 1. Therefore, for a specific structure with given parameters, we can always find a proper deviation ω to compensate the mismatch.

The condition S21.2 describes the requirement of the system symmetry, where γ_1 and γ_2 are radiation decay rates of port 1 (resonances at $-k_x$) and port 2 (resonances at k_x), respectively, shown in Fig. 1d in the main text. Obviously, condition S21.2 asks the system must have the same decay rates from the two radiation channels at $\pm k_x$, which can be fulfilled automatically by preserving the in-plane C_2 symmetry (two-fold in-plane rotational symmetry) or the z-mirror symmetry (vertical mirror symmetry, denoted as σ_z). Because for the z-mirror symmetry, there have $\gamma_1 = \gamma_3, \gamma_2 = \gamma_4$. Otherwise, reciprocity then requires that $\gamma_1 + \gamma_3 = \gamma_2 + \gamma_4$, so that $\gamma_1 = \gamma_2 = \gamma_3 = \gamma_4$. In other words, for system with C_2 in-plane symmetry or z-mirror symmetry, the condition S21.2 holds for any k_x. In this case, the DoFs of singularity is 1 (determined from the condition S21.1), and in 2D parameter space (ω, k_x), the trajectories of transmission singularity must be a curve close to the energy band: for any k_x, condition S21.2 and S21.3 automatically hold, and a proper frequency deviation ω is determined from the condition S21.1, shown in Fig. 1d in the main text.

However, such a nodal line can't provide the isolated singularity to generate a STOV. To achieve that, we break the in-plane C_2 symmetry and z-mirror symmetry. As a result, the condition S21.2 doesn't hold constantly. Since both γ_1 and γ_2 are real numbers, the DoF of condition S21.2 turns to be 1, too. Therefore, without the C_2 symmetry and z-mirror symmetry, considering both condition S21.1 and S21.2, the overall DoFs of the transmission singularity should be 2, and thus in 2D parameter space (ω, k_x) we can only find one point satisfying all the conditions: that's the STOV we want. Notice that the condition S21.2 holds at Γ point ($k_x = 0$) due to the protection of reciprocity even without the C_2 in-plane symmetry and z-mirror symmetry. Also, the break of C_2 symmetry and z-mirror symmetry ruin the symmetry-protected BIC at Γ point, open the vertical channel. Therefore, the STOV usually appears at Γ point without C_2 symmetry and z-mirror symmetry.

Section 3: The impact of the non-radiative loss

To best capture the nature of realistic samples, we take the non-radiative loss γ_{nr} that is contributed by absorption and scattering into account. Similarly, the transmission coefficient can be written as:

$$S_{14} = \frac{-t(\omega-\omega_0) \pm \sqrt{4r^2t^2\gamma_2\gamma_4 - (\gamma_1 - r^2\gamma_2 - t^2\gamma_4)^2}/t}{j(\omega-\omega_0)+\gamma_2+\gamma_4+\gamma_{nr}} + j\frac{t(\gamma_2-\gamma_4+\gamma_{nr}) - (\gamma_1 - r^2\gamma_2 - t^2\gamma_4)/t}{j(\omega-\omega_0)+\gamma_2+\gamma_4+\gamma_{nr}} \quad \text{(S22)}$$

Here, the necessary condition of zero-transmission in the ω-k_x space is also derived from the $\mathrm{Re}[S_{14}] = \mathrm{Im}[S_{14}] = 0$ as,

$$\begin{cases} (\omega-\omega_0)^2 = \dfrac{4r^2\gamma_2\gamma_4}{t^2} - (\gamma_2 - \gamma_4 + \gamma_{nr})^2 \\ \gamma_2 - \gamma_1 + t^2\gamma_{nr} = 0 \\ t \neq 0 \end{cases} \quad \text{(S23)}$$

We readily obtain that, for a nanograting breaking C_2 symmetry and z-mirror symmetry, the zero-valued singularity still exists in the ω-k_x space but it slightly deviates from the BZ center due to perturbation by non-radiation loss.

Section 4: Reference pulse characterization

Our reference pulse was obtained through spectral shaping of the initial femtosecond pulse with a 10 nm bandwidth Gaussian filter. Since there are virtually no transmissive optical components in the reference arm (only the two beam splitters with a low group delay dispersion of < 20 fs^2 at 800 nm, UFBS5050 from Thorlabs), the reference pulse remains nearly transform-limited and de-chirped (~80 fs) before the interferometric measurements. To further confirm this, we comprehensively characterized the reference pulse using a commercial frequency-resolved optical gating (FROG) system (FROGscan, Mesaphtonics). As shown in Fig. S3a, the pulse width of the reference pulse (blue solid line) is ~80 fs and is very close to a transform-limited pulse (red dashed line); its phase (green solid line) is flat, indicating no noticeable chirp. We also measured the spectrum and phase of the reference pulse (Fig. S3b), which shows that its spectrum (red solid line) is very close to a Gaussian distribution, and the corresponding phase (green solid line) is also flat. Similarly, the measured FROG trace shown in Fig. S3c indicates that the reference pulse does not possess a noticeable chirp. Therefore, the reference pulse we used will not significantly affect the reconstruction of the intensity and phase of the generated STOV.

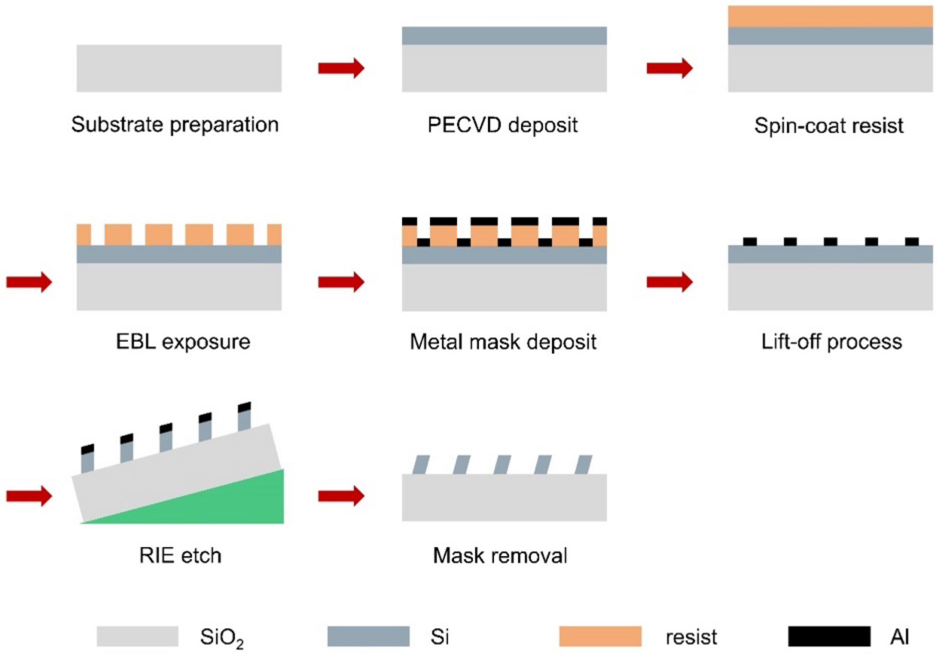

Fig. S1 | Step-by-step flow chart of the fabrication process. PECVD, plasma-enhanced chemical vapor deposition; EBL, electron-beam lithography; RIE, reactive ion etching.

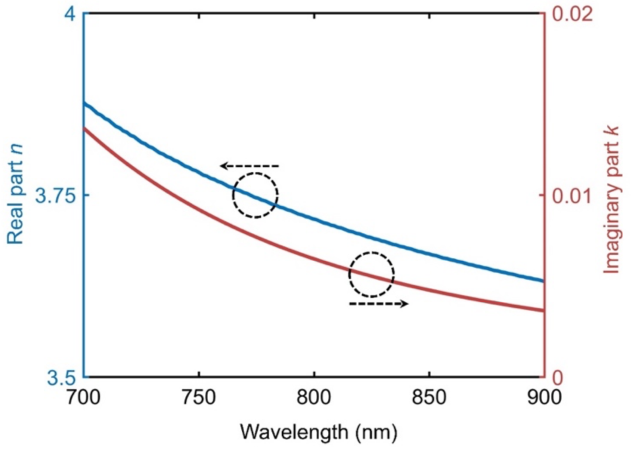

Fig. S2 | Refractive index of amorphous silicon. The real and imaginary part of the refractive index of α-Si (thickness ≈ 150 nm), deposited on a glass substrate, and measured using spectroscopic ellipsometry.

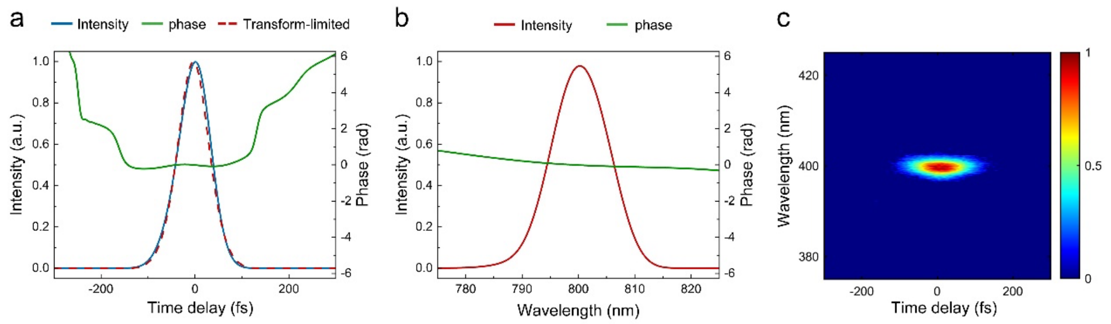

Fig. S3 | FROG measurement results of the reference pulse. a, Temporal intensity (blue solid line) and phase (green solid line) distribution. The red dashed line represents the transform-limited pulse. **b,** Spectral intensity (red solid line) and phase (green solid line) distribution. **c**, Measured FROG trace.

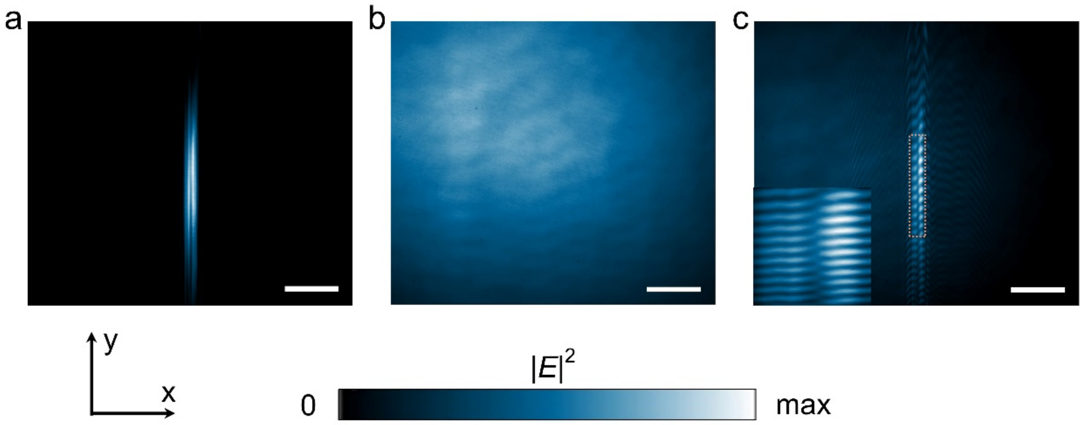

Fig. S4 | Spatial profiles of pulse light. **a**, Signal pulse. **b**, Reference pulse. **c**, Interference pulse. The insert: zoomed version of the orange dashed rectangular region. Scale bar: 1 mm.

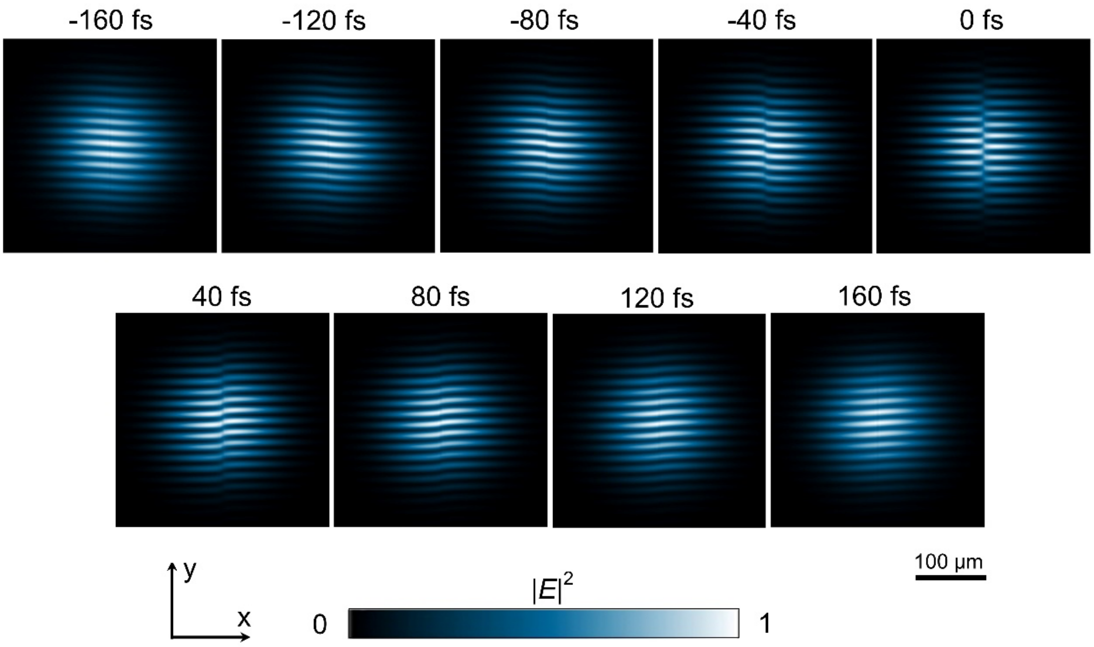

Fig. S5 | Theoretical fringe patterns in the x-y plane at various temporal locations of the STOV.

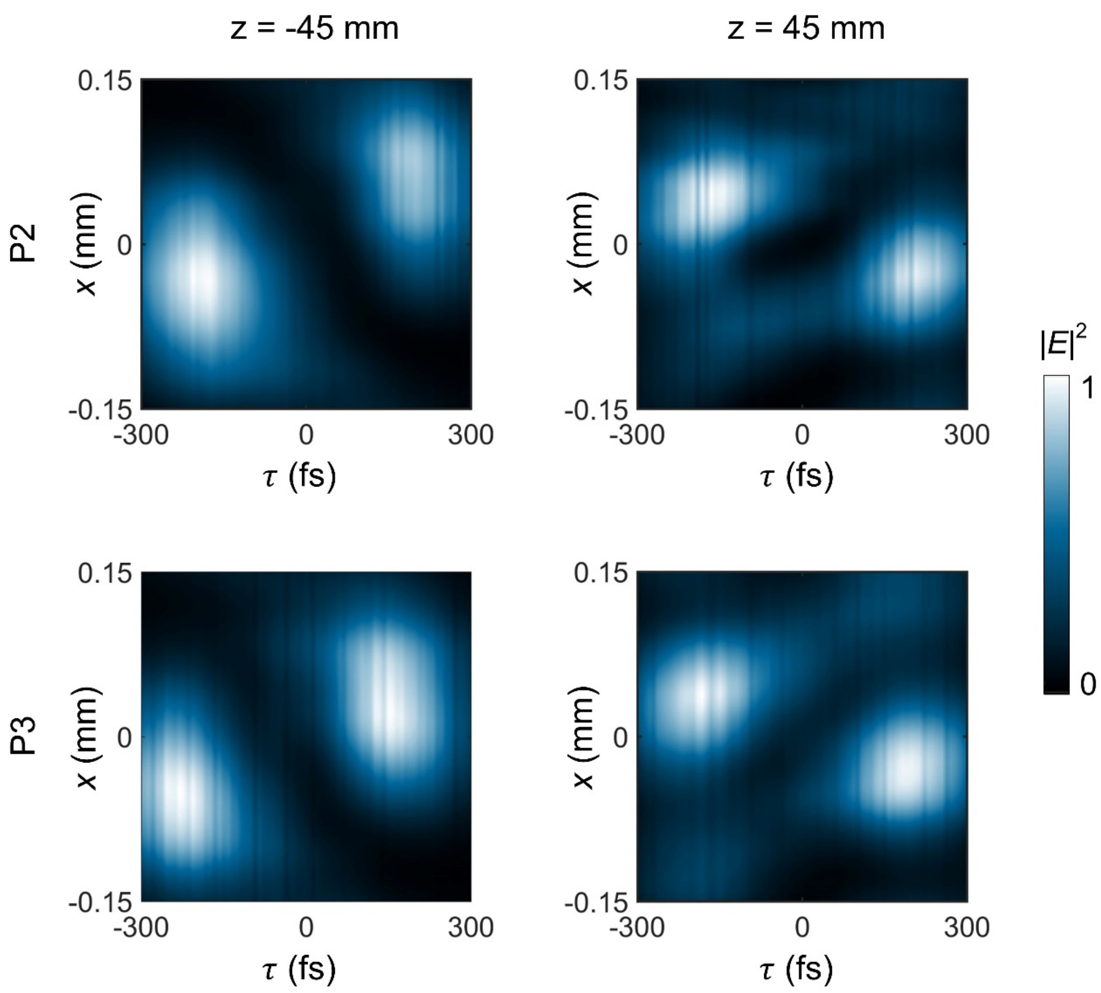

Fig. S6 | The reconstructed intensity distributions of the STOV at the locations of *z*=-45 mm and *z*=45 mm. The first and second rows present the dynamic evolution characteristics of the STOV generated at positions P2 and P3 of the sample, respectively.

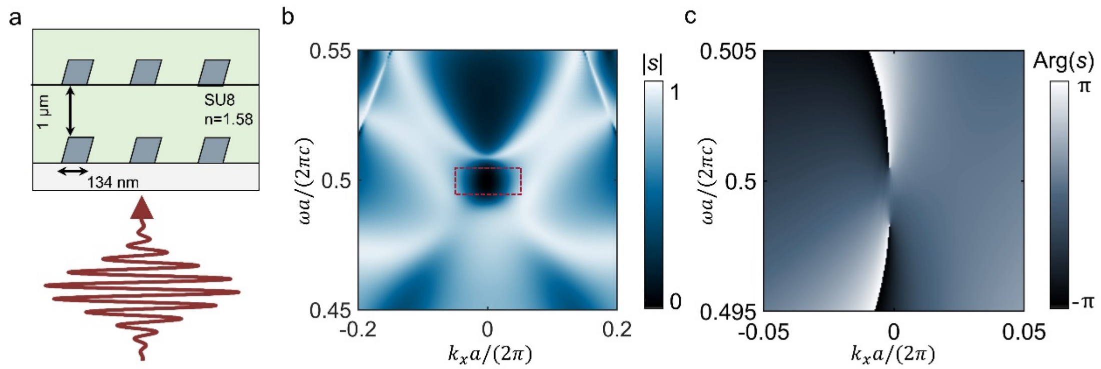

Fig. S7 | The construction of higher-order topological charges. a, Schematic diagram of the double-layer slanted grating. **b**, Simulated transmission coefficient distribution of the double-layer slanted grating. **c**, Phase distribution (topological charge *l*=2) corresponding to the red rectangle region of penal **b**.

Supplementary references

[1] S. Fan and J. D. Joannopoulos, Analysis of guided resonances in photonic crystal slabs. *Phys. Rev. B* **65**, 235112 (2002).

[2] S. Fan, W. Suh, and J. D. Joannopoulos, Temporal coupled-mode theory for the Fano resonance in the optical resonator. *J. Opt. Soc. Am. A* **20**, 569-572 (2003).

[3] W. Suh, Z. Wang, and S. Fan, Temporal Coupled-Mode Theory and the Presence of Non-Orthogonal Modes in Lossless Multimode Cavities. *IEEE J. Quantum Electron*. **40**, 1511-1518 (2004).

[4] H. Zhou, B. Zhen, C. W. Hsu, O. D. Miller, S. G. Johnson, J. D. Joannopoulos, and M. Soljačić, Perfect single-sided radiation and absorption without mirrors. *Optica* **3**, 1079-1086 (2016).

[5] W. Liu, B. Wang, Y. Zhang, J. Wang, M. Zhao, F. Guan, X. Liu, L. Shi, and J. Zi, Circularly polarized states spawning from bound states in the continuum. *Phys. Rev. Lett*. **123**, 116104 (2019).

[6] T. Yoda, and M. Notomi, Generation and Annihilation of Topologically Protected Bound States in the Continuum and Circularly Polarized States by Symmetry Breaking. *Phys. Rev. Lett*. **125**, 053902 (2020).

[7] Y. Zeng, G. Hu, K. Liu, Z. Tang, and C. Qiu, Dynamics of topological polarization singularity in momentum space. *Phys. Rev. Lett*. **127**, 176101 (2021).
